# Highly Sensitive Detection of Urea Using Si Electrolyte-Gated Transistor with Low Power Consumption

**DOI:** 10.3390/bios13050565

**Published:** 2023-05-22

**Authors:** Wonyeong Choi, Bo Jin, Seonghwan Shin, Jeonghyeon Do, Jongmin Son, Kihyun Kim, Jeong-Soo Lee

**Affiliations:** 1 Department of Electrical Engineering, Pohang University of Science and Technology (POSTECH), Pohang 37673, Republic of Korea; pathfinder@postech.ac.kr (W.C.); ssh3290a@postech.ac.kr (S.S.); toaru124@postech.ac.kr (J.D.); jmson@postech.ac.kr (J.S.); 2 Zhejiang RockerStone Electronics Technology Co., Ltd. (Defeng Electronic Technology), Jiaxing 314000, China; jinshengzhi1986@sina.com; 3 Division of Electronics Engineering, Jeonbuk National University, Jeonju 54896, Republic of Korea; kihyun.kim@jbnu.ac.kr

**Keywords:** biologically active field-effect transistor, electrolyte-gated transistor, high sensitivity, low power consumption, urea detection

## Abstract

We experimentally demonstrate Si-based electrolyte-gated transistors (EGTs) for detecting urea. The top-down-fabricated device exhibited excellent intrinsic characteristics, including a low subthreshold swing (*SS*) (~80 mV/dec) and a high on/off current ratio (~10^7^). The sensitivity, which varied depending on the operation regime, was analyzed with the urea concentrations ranging from 0.1 to 316 mM. The current-related response could be enhanced by reducing the *SS* of the devices, whereas the voltage-related response remained relatively constant. The urea sensitivity in the subthreshold regime was as high as 1.9 dec/pUrea, four times higher than the reported value. The extracted power consumption of 0.3 nW was extremely low compared to other FET-type sensors.

## 1. Introduction

Urea is a crucial biomarker for diagnosing various malfunctions in the human body. High urea levels in the blood can indicate conditions such as indigestion, kidney malfunction, renal failure, urinary tract obstruction, and gastrointestinal bleeding. In contrast, its low levels can indicate hepatic failure, nephritic syndrome, and cachexia [1]. The urea concentration (pUrea = $-\log_{10}\left[\mathrm{Urea}\right]$) in human blood ranges from 2.1 to 2.6 (3.5 mM to 7.5 mM).

Common methods used to analyze pUrea in patients include colorimetric and spectrometric techniques [2,3,4,5,6,7]. Colorimetric methods involve measuring the color changes using diacetyl monoxime, gold nanoparticles, polydopamine nanoparticles, and pH-sensitive hydrogels [2,3,4]. Spectrometric methods involve characterizing the fluorescence intensities of pH-sensitive dyes, gold nanoclusters, and quantum dots [5,6,7]. However, these optical-based techniques are time-consuming and require expensive equipment and skilled experts. To overcome the drawbacks above, electrochemical biosensors have been introduced.

Electrochemical biosensors have advantages such as fast response time, cost effectiveness, portability, and so on [8,9]. In order to further enhance their efficacy, improving key sensing parameters such as sensitivity, selectivity, and response time is of utmost importance. Recently, various types of transistors including ion-sensitive field-effect transistors (ISFETs) and biologically active FETs (BioFETs) have been developed to detect urea [10,11,12,13]. Nanostructure FET sensors have high sensitivity and can provide real-time and label-free detection [14,15]. However, the small sensing area of these sensors can limit the receptor density, resulting in insufficient output signals and significant device-to-device variations. Extended-gate FETs (EGFETs) are another type of ISFET consisting of a conventional FET and a separated sensing membrane connected to the gate [16,17]. However, the inherent interface between the gate and membrane generates additional parasitic capacitance and resistance, which worsens the sensitivity and reproducibility. More recently, electrolyte-gated FETs (EGTs) that use a functionalized gate electrode as the sensing surface have been developed [18,19,20,21,22]. The larger gate area, typically one order of magnitude larger than the channel area, is beneficial to achieve higher receptor density, thus enhancing output signals and reducing performance variations, which is crucial for the commercialization of BioFETs.

Herein, we investigated the electrical responses of Si-based EGTs for detecting urea. The device was fabricated using microfabrication technology. The Ag gate was functionalized with urease, and the current-voltage characteristics were experimentally measured at different pUrea values. The sensitivity and the limit of detection were analyzed in the subthreshold regime. Additionally, interference tests using typical biomolecules found in human blood were performed to evaluate the selectivity of the EGTs for detecting urea.

## 2. Materials and Methods

### 2.1. Material Preparation and Electrical Characterization

Urease from Jack Beans (Type III, powder, 20,000 units/g), urea (molecular biology grade, powder), phosphate-buffered saline (PBS, pH 7.4), (3-amino-propyl) triethoxysilane (APTES, 99%), glutaraldehyde (50%), glucose, ascorbic acid (AA), KCl, and anhydrous ethanol (200 proof, 99.5%) were purchased from Sigma-Aldrich (Burlington, VT, USA).

Prior to the experiments, a urea solution was prepared by dissolving urea powder in a 1 × PBS solution of pH 6. To test the selectivity of the device, other biomolecules such as glucose, AA, and KCl were also dissolved in the 1 × PBS solution with a pH of 6. The electrical characteristics of the device were measured using a semiconductor parameter analyzer (Keithley 4200, Keithley, Solon, OH, USA). The gate voltage (*V_G_*) was applied in increments of 50 mV through a buffer solution, while the drain current (*I_D_*) was measured with a fixed drain voltage (*V_D_*) of 0.1 V. The source and body voltages (*V_S_* and *V_B_*) were set to 0 V. *I_D_* was limited to 10^−7^ A to prevent the degradation of the device. The *I_D_*−*V_G_* characterizations were performed after exposing the target solution of 20 μL for 10 min.

### 2.2. Fabrication of EGTs

**Figure 1:**
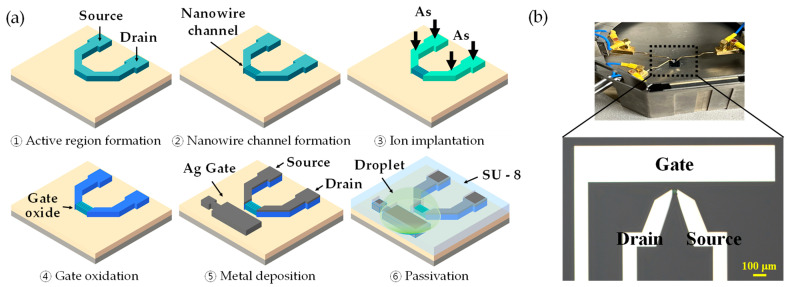
(**a**) Fabrication process flow of the Si-based EGT. (**b**) An optical image of the fabricated EGT. The area of the gate electrode is 1600 μm × 300 μm. The channel between the source and drain consists of 20 parallel nanowires with the length of 10 μm.

The EGTs were fabricated using a top-down method (Figure 1a) on a silicon-on-insulator wafer (p-type, 10 Ω∙cm, (100)) with a 140 nm-thick top-Si layer and 400 nm-thick buried oxide layer as the substrate material. The top Si layer was thinned to 100 nm using thermal oxidation to ensure the uniform doping of deep Si during ion implantation. The active region, consisting of the source, drain, and channel, was formed using an I-line stepper and an inductively coupled plasma reactive-ion etching (ICP-RIE) process. Using electron-beam lithography and ICP-RIE etching, the channel region was then patterned into nanowires with a width of 50 nm, 80 nm, and 110 nm, respectively. Arsenic ions (5 × 10^15^/cm^2^, 60 keV) were implanted into the source and drain regions, followed by rapid thermal annealing (RTA) at 1000 °C for 20 s. A 5 nm-thick SiO_2_ gate insulator was then thermally grown in a furnace at 800 °C for 5 min. Contact electrodes and transmission lines were formed using Ag/Ti (500 nm/50 nm) layers deposited via an e-beam evaporator and lift-off process. Finally, a 2 μm-thick SU-8 layer was passivated on the surface for electrical isolation, excluding the channel, gate electrode, and contact pads (Figure 1b).

### 2.3. Functionalization of EGTs

As a urea receptor, the urease was immobilized on the gate area. The gate electrode was first treated with UV/ozone for 90 s under a light intensity of 200 µW/cm^2^ to generate hydroxyl groups (OH^−^). The surface was then exposed to vaporized APTES at 55 °C for 1 min, rinsed with anhydrous ethanol to remove unbound APTES molecules, and dried using N_2_ blowing. The devices were then immersed in a glutaraldehyde solution (2.5 %, 1 × PBS, pH 7.4) for 90 min, washed with 1 × PBS and DIW, and dried with N_2_ blowing. Finally, the devices were exposed to a urease solution (10 mg/mL, 1 × PBS, pH 7.4) for 18 h in a humid environment at 4 °C, followed by rinsing with 1 × PBS and DIW and drying with N_2_ blowing.

**Figure 2:**
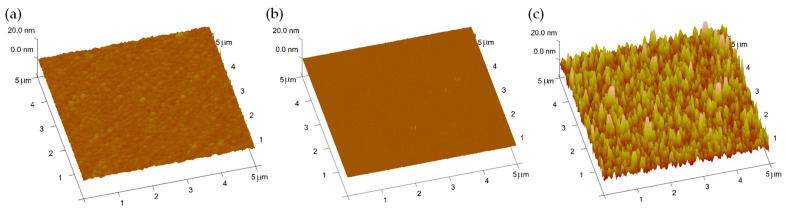
3D surface plot of the AFM analysis for the (**a**) bare Ag, (**b**) Ag after APTES/GA treatment, and (**c**) urease functionalized Ag surface.

The urea functionalization on the Ag gate was verified using atomic force microscopy (AFM, VEECO, New York, NY, USA), as shown in Figure 2. The average roughness values were determined to be 0.7 nm for the bare Ag surface, 0.13 nm after APTES/GA treatment, and 4.2 nm following the immobilization of urease, respectively. The reduction in roughness observed after APTES/GA treatment can be attributed to the effective filling of APTES molecules within the Ag grain boundaries [23].

## 3. Results and Discussion

### 3.1. Intrinsic Electrical Characteristics

**Figure 3:**
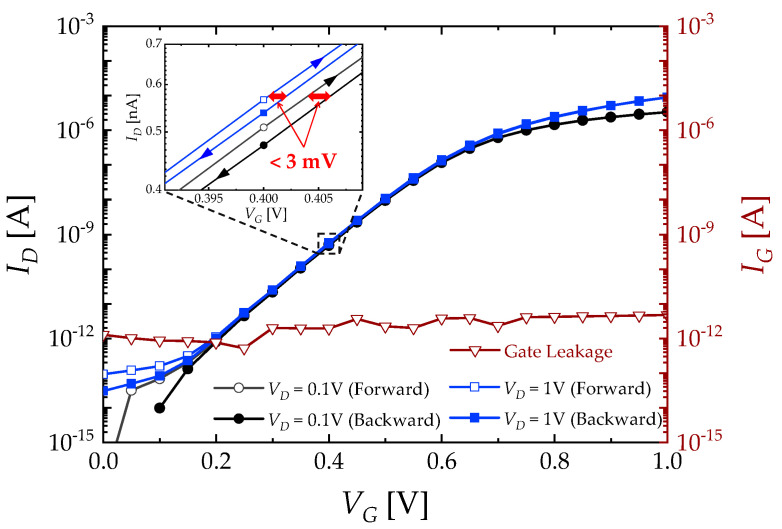
Intrinsic characteristics of the fabricated EGT are shown in the log-scale *I_D_*−*V_G_* curve under forward and backward sweeps for *V_D_* = 0.1 and 1 V (left axis) and in the log-scale *I_G_*−*V_G_* curve (right axis). Inset provides an enlarged *I_D_*−*V_G_* curve with hysteresis characteristics.

Figure 3 shows the intrinsic transfer curve (*I_D_* vs. *V_G_*) and gate leakage current (*I_G_*) of the EGT device. It exhibits excellent n-type characteristics including a low subthreshold swing (*SS*) of ~80 mV/dec, high on/off current ratio (*I_ON_*/*I_OFF_*) of ~10^7^, and low threshold voltage (*V_TH_*) of ~0.65 V. The low leakage current (<10 pA) and negligible hysteresis (inset of Figure 3) guarantee a reliable and reproducible operation during sensing responses.

### 3.2. Sensing Characteristics

**Figure 4:**
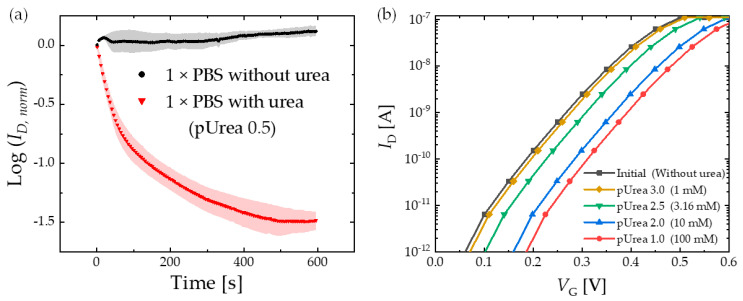
(**a**) Real-time monitoring of the normalized *I_D_* (*I_D, norm_*) of the EGT exposed to 1 × PBS with and without urea (pUrea 0.5) at a fixed *V_G_* of 0.3 V. *I_D, norm_* refers to the ratio of *I_D_* to *I_D_*_0_, where *I_D_*_0_ represents the initial *I_D_* measured at time = 0 s. (**b**) Representative *I_D_*−*V_G_* curve of the EGT with varying concentrations, with a current compliance of 0.1 µA applied.

Figure 4a shows the current monitoring result for 1 × PBS with and without urea (pUrea 0.5) at a fixed *V_G_* of 0.3 V. Five devices were used to obtain each data point, and the average value and 1σ of those measurements are plotted. Over time, *I_D_* continuously decreased for the urea solution, whereas it remained constant for 1 × PBS. Since the response for the urea saturated within the first 10 min of exposure, 10 min exposure time was used for all experiments. The urea in a solution reacts with the urease on the Ag surface to produce the OH^−^ ions and to increase the pH value.

Figure 4b shows the change in the transfer curve as the device is exposed to different pUrea values. The initial state denotes the *I_D_*−*V_G_* curve without urea. An increase in the urea concentration or a decrease in the pUrea value caused the curve to shift toward a positive *V_G_* direction.

The current-related response (*R_I_*) is defined as follows [24,25]:

(1) $$R_{I}=\frac{I_{D0}-I_{D1}}{I_{D1}},$$

where *I_D_*_0_ and *I_D_*_1_ represent drain currents at a fixed *V_G_*_0_ before and after the reaction, respectively. *V_G_*_0_ of 0.3 V was selected to calculate *R_I_* from the data presented in Figure 4b.

The voltage-related response (*R_V_*) is defined as follows [26]:

(2) $$R_{V}=V_{G1}-V_{G0},\;$$

where *V_G_*_0_ and *V_G_*_1_ represent gate voltages at a fixed *I_D_*_0_ before and after the reaction, respectively. The *I_D_*_0_ of 3 nA and *V_G_* of 0.3 V were chosen because the current was significantly higher than the noise level (~1 pA), and it ensured the device was operated in the subthreshold regime below the *V_TH_* of 0.65 V.

To achieve a high sensitivity, FET-based biosensors should be operated in the subthreshold regime [27,28], where *I_D_* and *SS* are defined as follows [29]:

(3) $$I_{D}=\mu_{n}\left(C_{ox}+C_{it}\right)\frac{W}{L}{\left(\frac{kT}{q}\right)}^{2}\left(1-e^{-\frac{qV_{D}}{kT}}\right)e^{\frac{q\left(V_{G}-V_{T}\right)}{nkT}}\;;$$

(4) $$SS\equiv \frac{\partial V_{G}}{\partial \log I_{D}}=\frac{kT}{q}\ln\left(10\right)\left[1+\frac{C_{d}+C_{it}}{C_{ox}}\right],$$

where *μ_n_* is the electron mobility; *C_ox_* is the oxide capacitance; *C_it_* is the interface state capacitance; *W* is the channel width; *L* is the channel length; *k* is the Boltzmann constant; *T* is the temperature; *q* is the electron charge; and *C_d_* is the depletion capacitance in the channel.

*R_I_* at a fixed *V_D_* can also be expressed as follows:

(5) $$R_{I}=\frac{I_{D0}}{I_{D1}}-1=\frac{e^{\frac{ln\left(10\right)\left(V_{G}-V_{TH0}\right)}{SS}}}{e^{\frac{ln\left(10\right)\left(V_{G}-V_{TH1}\right)}{SS}}}-1=e^{\frac{ln\left(10\right)\Delta V_{TH}}{SS}}-1=e^{\frac{ln\left(10\right)R_{V}}{SS}}-1,$$

where *V_TH0_* and *V_TH1_* represent threshold voltages before and after the reactions, respectively. Therefore, *R_I_* can exponentially increase as *R_V_* increases.

**Figure 5:**
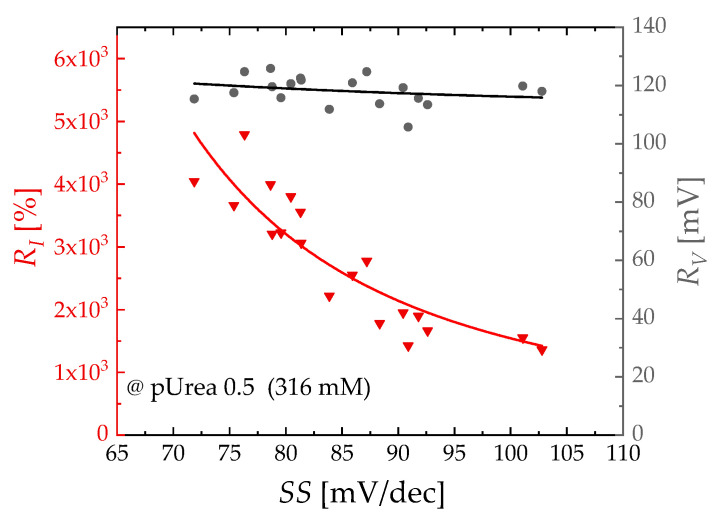
*R_I_* vs. *SS* (triangle symbol, left axis) and *R_V_* vs. *SS* (circle symbol, right axis) curves at pUrea = 0.5. Solid curves represent the exponential fitted curves for *R_I_* and *R_V_*, respectively.

Figure 5 illustrates the dependence of *R_I_* and *R_V_* with respect to the *SS* value at a pUrea of 0.5. The extracted *R_V_* was approximately 120 mV, displaying a consistent behavior across different *SS* values. In contrast, *R_I_* was inversely proportional to *SS* values and decreased as *SS* increased. The exponential calibration curve of *R_I_* and *R_V_* was obtained as *R_I_* = 100 × (e ^ln(10) × 122/*SS*^ − 1) and *R_V_* = 61.2 × e ^−*SS*/36.0^ + 112.

**Figure 6:**
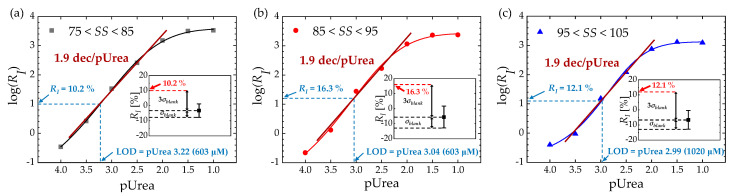
log(*R_I_*) vs. pUrea and LOD extraction for (**a**) 75 < *SS* < 85, (**b**) 85 < *SS* < 95, and (**c**) 95 < *SS* < 105. Solid lines represent logistic fitted lines for each range of *SS*. Insets: *R_I_* for the blank sample (1 × PBS without urea) and *R_I_* at the LOD using the three–sigma method for each range of *SS*.

Figure 6 shows the relationship between the *R_I_* and pUrea for different *SS* values. The EGTs with low *SS* values (75 < *SS* < 85) exhibit a saturated *R_I_* of 3.3 × 10^3^ (%) at a pUrea of 1.0. Conversely, EGTs with higher *SS* values (95 < *SS* < 105) exhibit a lower saturated *R_I_* of 1.3 × 10^3^ (%) at the same pUrea value. As determined by the slope of the logistic fitted line of *R_I_*, the consistent urea sensitivity of 1.9 dec/pUrea is achieved across all *SS* values, which is more than four times higher than the previous results (Table 1). The dynamic range, defined as the difference between 10% and 90% of the maximum sensitivity, is observed to be between pUrea 2.0 and pUrea 3.4 regardless of *SS* values, which fully encompasses the clinical range of human urea. The limit of detection (LOD) of *R_I_*, determined using the 3–σ method from the logarithmic trend line [30,31], is as low as pUrea 3.22 for 75 < *SS* < 85, pUrea 3.04 for 85 < *SS* < 95, and pUrea 2.99 for 95 < *SS* < 105.

**Figure 7:**
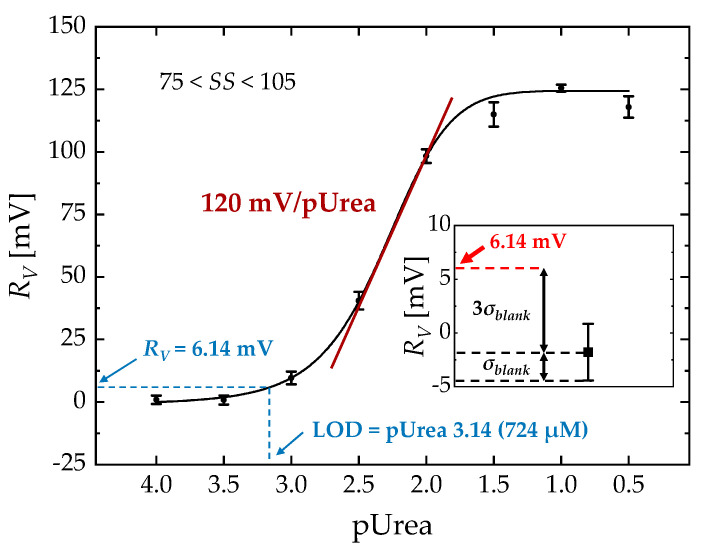
*R_V_* vs. pUrea at a fixed *I_D_*_0_ of 3 nA. A solid curve represents a logistic fitted line. Inset: *R_V_* for the blank sample (1 × PBS without urea) and *R_V_* at the LOD using the three–sigma method.

Figure 7 shows the relationship between *R_V_* and pUrea over the whole range of *SS* (75 < *SS* < 105). Each point represents the average of five different devices. A dynamic pUrea range of 1.8–2.9 was obtained. The urea sensitivity extracted from the *R_V_* curve was 120 mV/pUrea, with a LOD of pUrea 3.14.

Power consumption is a crucial factor for portable biosensing applications. The calculated power consumption with *V_D_* = 0.1 V and *I_D_* = 3 nA is significantly lower than that of other FET-type biosensors due to the operation in the subthreshold regime. Table 1 compares the sensing performance of the EGT with that of previously reported FET-type sensors.

### 3.3. Selectivity Test

**Figure 8:**
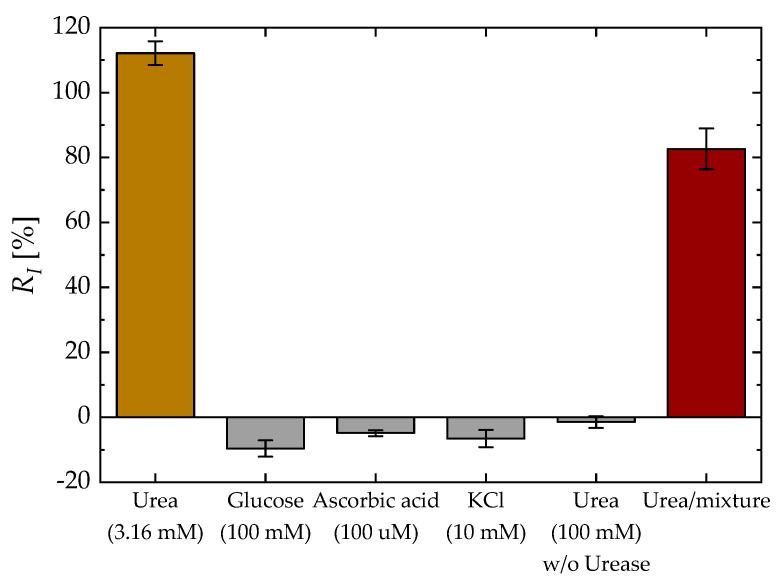
Control experiments: *R_I_* for urea (3.16 mM, pUrea 2.5), glucose (100 mM), AA (100 μM), KCl (10 mM), urea (100 mM) without urease treatment, and the urea/mixture. The urea/mixture sample includes urea (3.16 mM), glucose (100 mM), AA (100 μM), and KCl (10 mM).

Figure 8 shows the *R_I_* of various common interferents found in human blood including glucose (100 mM, 1 × PBS), AA (100 μM, 1 × PBS) and KCl (10 mM, 1 × PBS), and *R_I_* of urea (100 mM) with unmodified EGT (without urease) to demonstrate the lack of nonspecific binding of the device. All devices except the unmodified EGT were functionalized using the same method described in Section 2.3. Each data point corresponds to the average measurement obtained from five devices. The interference response for individual ingredients was found to be minimal, with less than a 10% change compared to the signal observed with urea at a concentration of 3.16 mM. A negligible *R_I_* of the unmodified sample indicates that there is no nonspecific binding between the urea and a Ag surface. Although the *R_I_* of the urea/mixture sample was reduced due to the opposite signal direction of the interferents compared to urea, it was still detectable at a sufficient level. This suggests the stability of the EGT sensing performance and the minimal impact of interfering ions on its urea response.

## 4. Conclusions

We investigated the label-free sensing response of urea using Si-based EGTs. The device was fabricated using a top-down microfabrication technique and operated in the subthreshold regime to enhance the sensitivity. The EGTs with a low *SS* could further increase the current-related responses. The urea sensitivities determined from *R_I_* and *R_V_* were as high as 1.9 dec/pUrea and 120 mV/pUrea, respectively. The calculated power consumption was as low as 0.3 nW and three orders of magnitude lower compared to previously reported results. In addition, the extracted dynamic range fully covered the human clinical range of urea. These results suggest that Si-based EGTs have significant potential for clinically diagnosing urea-related diseases.

## Figures and Tables

**Table 1:** Performance comparison of FET-type urea biosensors.

FET Type	Dynamic Range (pUrea)	Urea Sensitivity (*R_I_*) (dec/pUrea)	Urea Sensitivity (*R_V_*) (mV/pUrea)	Power Consumption	Ref
AlGaN/GaN ion-sensitive FET	1.6–3.4 (*R_I_*)	0.24 (Linear regime)	–	6 W	[10]
ZnO nanorod FET	–	0.27 (Linear regime)	–	–	[11]
EGFET Membrane: ITO layer	–	–	62.4 (Linear regime)	500 nW	[32]
EGFET Membrane: SnO_2_:F layer	1–3.1 (*R_V_*)	0.42 (Linear regime)	109 (Linear regime)	25 mW	[12]
Si-based EGT	**2.0–3.4 (*R_I_*)** **1.8–2.9 (*R_V_*)**	**1.9** **(SubV_TH_ regime)**	**120** **(SubV_TH_ regime)**	**0.3 nW**	**This work**

## Data Availability

Not applicable.
